# Artificial intelligence to enhance prehospital stroke diagnosis and triage: a perspective

**DOI:** 10.3389/fneur.2024.1389056

**Published:** 2024-05-02

**Authors:** Zoe C. Wolcott, Stephen W. English

**Affiliations:** Department of Neurology, Mayo Clinic, Jacksonville, FL, United States

**Keywords:** prehospital stroke, artificial intelligence, mobile stroke unit, telemedicine, computer vision, stroke triage, LVO stroke

## Abstract

As health systems organize to deliver the highest quality stroke care to their patients, there is increasing emphasis being placed on prehospital stroke recognition, accurate diagnosis, and efficient triage to improve outcomes after stroke. Emergency medical services (EMS) personnel currently rely heavily on dispatch accuracy, stroke screening tools, bypass protocols and prehospital notification to care for patients with suspected stroke, but novel tools including mobile stroke units and telemedicine-enabled ambulances are already changing the landscape of prehospital stroke care. Herein, the authors provide our perspective on the current state of prehospital stroke diagnosis and triage including several of these emerging trends. Then, we provide commentary to highlight potential artificial intelligence (AI) applications to improve stroke detection, improve accurate and timely dispatch, enhance EMS training and performance, and develop novel stroke diagnostic tools for prehospital use.

## Introduction

Despite significant advancements in stroke treatments including thrombolytic therapy and mechanical thrombectomy for patients with large vessel occlusion (LVO), stroke remains a leading cause of long-term disability (1). These therapies operate on the principle that recanalization of occluded arteries reduces brain injury, but treatment is highly time dependent (2). Moreover, access to stroke treatment remains limited, as few hospitals have resources to deliver these interventions. Therefore, healthcare systems have organized such that hospitals with primary stroke center (PSC) certification can administer thrombolytic treatment, whereas only comprehensive stroke centers (CSC) are accredited to perform mechanical thrombectomy in patients with LVO stroke (3, 4). This regional distribution increases pressure on our Emergency Medical Services (EMS) to ensure patients are directed to the appropriate hospital. The stakes are high, emphasized by a recent US stroke registry reported average door-in-door-out time of 174 min for patients required transfer to a higher level of care, and up to one-third of patients with LVO become ineligible for thrombectomy due to delays in transfer to a comprehensive stroke center (3–7). Therefore, EMS personnel have become crucial stakeholders in the stroke care continuum.

## Current state

Prehospital EMS systems have adapted to the changing acute stroke treatment landscape. Emergency 9-1-1 dispatch protocols prioritize stroke calls as the highest acuity (8, 9). EMS prehospital notification facilitates early mobilization of stroke teams and downstream resources (9, 10). And scales designed to identify LVO stroke have been validated to enhance prehospital triage (11, 12). National recommendations call for an initial stroke screening tool, and if positive, an LVO screening tool in patients within 24 h of last known well. Recommendations call for bypass of primary stroke centers if the comprehensive stroke center is within 30 min for urban, 45 min for suburban, and 60 min for rural settings if the prehospital LVO screen is positive (11). Despite these changes, many stroke patients are not recognized in the prehospital setting. Contributing factors include inadequate training, limited EMS resources, high EMS personnel turnover, suboptimal scale accuracy, poor scale utilization, and inherent variability in presenting stroke symptoms.

Despite these shortcomings, there are reasons for optimism. First, incorporating prehospital LVO screening tools have demonstrated almost 3-fold higher thrombectomy rates for eligible patients presenting directly to thrombectomy-capable centers (4.8% without vs. 13.6% with LVO screening tool) (3, 12). And recently, mobile stroke units (MSU)—specialized ambulances equipped with CT, telemedicine, and stroke nurses—have demonstrated both faster treatment (10-fold increase in patients treated within 60 min of symptom onset) and an 8% increase in excellent outcomes 90 days—compared to conventional ambulance care (13, 14).

To date, the MSU model has experienced limited uptake, likely driven by several factors: (1) high MSU cost (both upfront and recurring costs), (2) poor reimbursement, and (3) logistical and bureaucratic challenges to integrate and scale within existing EMS infrastructure. Due to these challenges, several groups have explored ambulance-based telemedicine to bring vascular neurology expertise to the ambulance (11, 15); one group found that ambulance-based telemedicine was 100% accurate in predicting reperfusion candidates compared to 70% for the LVO scale (11, 15). This approach has key advantages, as it is more scalable due to lower upfront costs, can leverage existing ambulances, and can be leveraged for non-neurologic indications including trauma and cardiac emergencies.

## Future state: artificial intelligence to enhance prehospital stroke care

**Figure 1:**
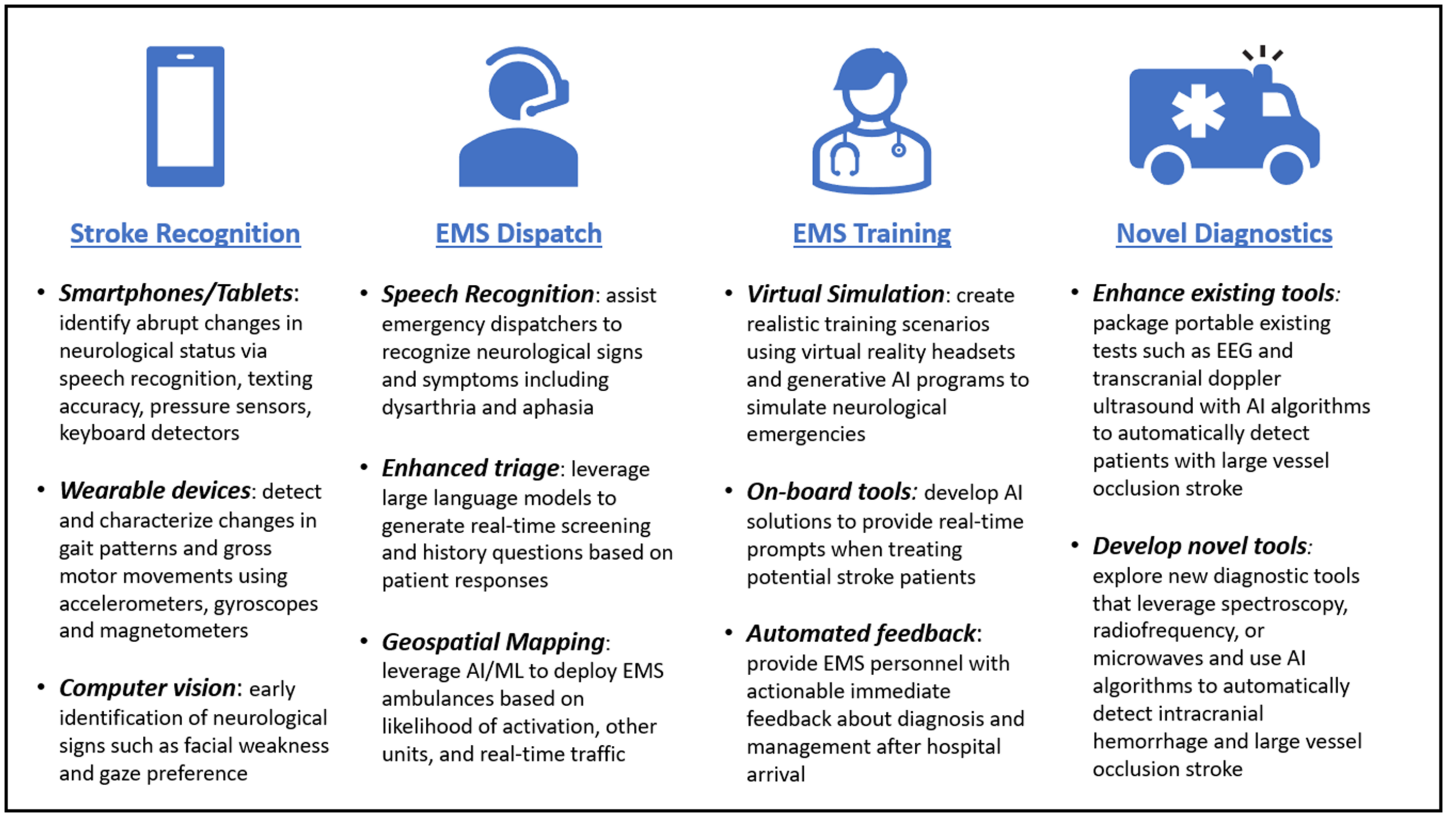
Overview of potential artificial intelligence applications to enhance prehospital stroke diagnosis and triage.

MSUs and telemedicine-enabled ambulances highlight how the paradigm shift for prehospital stroke care has promoted innovations to confront this emerging demand. We foresee the most impactful ways to minimize prehospital delays include improved public awareness, efficient and accurate emergency dispatch, enhanced paramedic evaluation and triage, and streamlined inter-facility workflows (16, 17). Future innovations in telemedicine and artificial intelligence (AI) will undoubtedly improve prehospital stroke diagnosis and triage and allow for more standardization in clinical practice. The authors provide insights into specific opportunities we believe AI applications will hold the most promise to enhance prehospital stroke care (Figure 1).

### Stroke recognition tools

Perhaps the most common reason for delays in stroke treatment can be attributed to delayed recognition of stroke symptoms by patients or families. AI solutions via wearable technologies and computer vision may help reduce this barrier by expediting EMS activation (18). Wireless sensors and wearable technologies including watches, fitness bands, and rings could be used to characterize limb motor deficits and gait patterns using accelerometers, gyroscopes, and magnetometers (18, 19). Smartphones, tablets, and computers can be turned into powerful screening tools by identifying sudden changes in motor function, speech, or language by incorporating artificial intelligence in touch screen, keyboard, and speech-to-text inputs through pressure sensors, keyboard detectors, and speech recognition software (18). This technology has the potential to alert individuals to the onset of stroke symptoms and hasten EMS activation (19, 20). For example, one recent AI algorithm reportedly reviewed 269 patients and found 97% accuracy in detection of facial asymmetry and 72% accuracy in detection of arm weakness (21, 22), and improvements in eye tracking may identify sudden changes in eye movements or unilateral gaze deviation, a dependable biomarker for LVO stroke (23, 24). In fact, observation of head and/or gaze deviation alone in telemedicine consultations has been shown to predict LVO with a sensitivity of 0.70 and specificity of 0.93, and MT necessity with a sensitivity of 0.86 and specificity of 0.90 (23). Incorporating automated detection of gaze preference, aphasia, neglect, and facial palsy into a single application may aid in early detection of acute strokes and identify patients with LVO strokes, facilitating pre-hospital triage to a comprehensive stroke center (25).

### EMS dispatch

Emergency dispatcher stroke recognition is associated with higher EMS utilization of stroke scales, EMS stroke identification, and faster response times (26–28). Despite specific stroke training, the overall accuracy of emergency dispatchers leaves room for improvement (26, 29). Several groups have investigated AI as a means to improve dispatch accuracy and resource allocation. One study reviewed the potential impact of automated speech recognition software on stroke recognition in Denmark, and found that it could improve stroke detection by 16% and increase thrombolysis use by 2% (30). Another study scrutinized the application of AI algorithms in ambulance dispatch protocols for motor vehicle accidents. Through predictive analytics, this study anticipated optimal deployment times and locations for ambulances to expedite response times (31). We envision a future state where similar AI strategies can be deployed to position ambulances based on factors such as time of day, regional demographics, and other conflicting emergency calls to ensure fast response.

### EMS personnel training and competency assessments

Generative AI and virtual reality may soon transform medical education through the development of immersive medical simulation experiences (32, 33). Stroke only accounts for approximately 2% of total EMS dispatches, and with high turnover and limited shifts, EMS personnel may lack appropriate training or experience to identify and triage stroke. While screening tools are helpful, specific simulation exercises leveraging virtual or augmented reality can provide structured educational experiences to augment training prior to real-world encounters. We envision specific software tailored to each healthcare provider role that fosters practice on simulated stroke patients in a “no-stakes” environment. These tools can provide real-time feedback to enhance retention and give EMS personnel valuable repetitions to improve performance. Similar training experiences have already been developed for surgical training and sepsis management (34, 35). Moreover, real-time AI-enhanced feedback could significantly enhance EMS provider training and competence by offering immediate insights during patient assessment, helping them to learn and adapt more effectively (30, 35, 36). This ongoing training can lead to improved performance, ensuring our EMS personnel are equipped to handle the complexities of stroke diagnosis. Nonetheless, it’s crucial to recognize that while AI can augment clinical scales and inform examinations, the inherent variability and frequency of stroke mimics leave room for error if triage decisions are solely reliant on clinical severity scores (34, 37).

### Intelligent telemedicine

Stroke neurologists were pioneering end-users of telemedicine, connecting with remote patients to provide real-time audio-video consultation in remote underserved locations for over a decade (38). The COVID-19 pandemic has accelerated the uptake of telemedicine, creating opportunities to explore AI applications to the video encounter including computer vision tools that can precisely measure human movements from videos and may allow for autonomous recognition of stroke symptoms (39). Clear use cases for image-based AI applications have already been reported in the fields such as cardiology, pathology, dermatology, and ophthalmology (40), but by applying deep-learning algorithms to live patient videos, we believe this may soon allow for the automated assessment of neurologic examinations such as the LVO stroke scales and the NIHSS. While these applications have not been tested in this clinical context, we envision the use of future computer vision applications, either alone or in conjunction with video telestroke services to more accurately recognize focal neurologic deficits and improve prehospital triage.

### Novel diagnostic tools

The ability to differentiate hemorrhagic stroke, non-LVO stroke, and LVO stroke remains a critical challenge in prehospital stroke care. AI integration with both existing and emerging technologies holds promise to develop cost-effective tools to assist in prehospital stroke diagnosis. Efforts in this arena are ongoing, including applications that harness electroencephalography (EEG) and transcranial doppler (TCD) to identify LVO. Both of these are attractive options due to their inherent portability, low-cost, and potential discriminatory ability to differentiate stroke syndromes. One study demonstrated an EEG cap during ambulance transportation can accurately differentiate LVO stroke, reporting a diagnostic accuracy of 91% and a positive likelihood ratio of 11.0 (41). Another study investigating a portable, automated TCD device and found the overall accuracy of LVO detection was 91% when combining two biomarkers. While these results demonstrated the potential value of AI to enhance existing point-of-care diagnostics, these devices have not been rigorously tested in a real-world prehospital setting yet and may not yet be ready for widespread use.

Advancements in AI have facilitated the development of novel diagnostic methods capable of swift detection and triage of stroke patients. Automated headsets have demonstrated potential in characterizing brain tissue characteristics by utilizing spectroscopy, radiofrequency, and microwave detection (42–44). AI algorithms applied in this context have been successful in differentiating patients with acute intracerebral hemorrhage and LVO stroke from healthy controls (20, 43, 44). One emerging example is a Volumetric Impedance Phase Shift Spectroscopy (VIPS) headset, a non-invasive tool that measures electrical impedance of different tissue types using electromagnetic waves. VIPS can detect lateralized changes associated with brain injury, and research suggests it can accurately identify patients with severe stroke (either LVO stroke or large ICH) with a 93% sensitivity and 87% specificity (43). Similar headsets are investigating the role of radiofrequency and low-energy microwave signals couple with machine learning algorithms to detect traumatic brain injury, intracranial hemorrhage, and LVO stroke, but results have not yet been published (44). With time, we believe these devices may provide scalable solutions to enhance existing regional stroke triage.

## Conclusion

As the landscape of acute stroke treatment continues to evolve, the significance of prehospital stroke recognition, accurate diagnosis, and effective triage has become increasingly valuable to ensuring optimal patient care. Current prehospital stroke triage tools range from basic stroke severity scales with limited accuracy to MSUs that can accurately diagnosis and treat patients with stroke prior to hospital arrival. Resource and technology limitations continue to hamper the growth of prehospital stroke management, but we believe several promising AI applications have the potential to address this need through greater recognition of stroke symptoms, improved dispatch, enhanced EMS training and feedback, and development of novel stroke diagnosis and triage tools.

## Data availability statement

The original contributions presented in the study are included in the article/supplementary material, further inquiries can be directed to the corresponding author.

## Author contributions

ZW: Writing – review & editing, Writing – original draft. SE: Writing – review & editing, Conceptualization, Supervision.
